# People, process, technology: a framework for clinical informatics fellowship applicants to evaluate programs

**DOI:** 10.1093/jamiaopen/ooag006

**Published:** 2026-01-20

**Authors:** Jared Silberlust, Priyanka Solanki, Jonathan Austrian, Paul Testa, Nicholas Genes

**Affiliations:** MCIT Department of Health Informatics, NYU Langone Health, New York, NY 10016, United States; MCIT Department of Health Informatics, NYU Langone Health, New York, NY 10016, United States; MCIT Department of Health Informatics, NYU Langone Health, New York, NY 10016, United States; MCIT Department of Health Informatics, NYU Langone Health, New York, NY 10016, United States; MCIT Department of Health Informatics, NYU Langone Health, New York, NY 10016, United States

**Keywords:** Clinical Informatics Fellowship, Graduate Medical Education, People Process Technology

## Abstract

**Objectives:**

To propose a structured framework for evaluating and comparing clinical informatics fellowship programs using the People, Process, and Technology (PPT) model.

**Materials and Methods:**

We adapted Leavitt’s organizational theory to create a three-pillar framework operationalized with features relevant to fellowship applicants and directors. We then applied this framework to a random sample of 18 program websites.

**Results:**

The PPT framework categorizes key fellowship characteristics into People (eg, mentorship, co-fellows, diversity), Process (eg, clinical duties, research emphasis, education), and Technology (eg, EHR systems, technical training, remote work). A visual grid illustrates variation in operational versus research focus and levels of mentorship. Website analysis revealed inconsistent transparency and detail.

**Discussion:**

The PPT framework provides a systematic, accessible approach for applicants to assess fellowship fit and for programs to communicate their strengths.

**Conclusion:**

Standardizing fellowship descriptions using the PPT model may improve alignment between applicant goals and program offerings, enhancing both the application process and training experience.

## Introduction

Since the launch of ACGME-accredited clinical informatics fellowship programs in 2014, the number of programs has grown to over 51 nationwide.^1^ This expansion has created both new opportunities and challenges for applicants and programs. Many applicants report spending interview days asking the same foundational questions—about mentorship, research versus operations, technical training, and clinical responsibilities—suggesting that such information is not readily available or standardized. As a result, valuable interview time is spent filling in basic gaps rather than engaging in deeper conversations about faculty alignment or project fit. Applicants also face uncertainty about how well programs will prepare them for various career paths. Alumni have reported asymmetric skill development—feeling highly trained in some domains (eg, operations) but underprepared in others (eg, AI, cybersecurity, data science). Others described gut-driven decision-making, choosing programs based on interpersonal “fit” or intuition rather than a systematic comparison—largely because elements like mentorship, technical exposure, and daily responsibilities were not clearly communicated. Without a transparent structure for comparing strengths, applicants often rely on these gut feelings rather than clear insights when choosing where to train.

Fellowship programs also seek more efficient ways to showcase their offerings in a competitive environment. Program directors often lack time or bandwidth to meet with every applicant before, during, and after interviews. Without a shared language or structure, it becomes difficult to communicate a program’s nuances in an accessible, comparable way. Applicants are left with an incomplete picture of daily life as a fellow—limiting their ability to make informed decisions. While it’s impossible to fully understand a role without lived experience, the clinical informatics fellowship community has room to augment the current, unstructured process with a simple framework.

We propose using the People, Process, Technology (PPT) framework—a model rooted in organizational change and systems thinking, originally popularized by Harold Leavitt’s Diamond Model in 1965^2^—as a structured way to present fellowship characteristics. The PPT framework has been widely adopted in healthcare to break down complex systems into understandable, actionable components. Across diverse settings—from electronic medical record (EMR) implementation in Kenyan public hospitals^3^ to evidence-based practice in high-income health systems^4^—the PPT framework has served as a guiding structure to align organizational efforts, clarify stakeholder roles, and support sustainable change. In population health management^5^ and HIT modernization initiatives,^6^ it has helped balance human-centered needs with technical and procedural demands. Moreover, its emphasis on knowledge sharing and continuous learning has made it a valuable model for transforming global health systems.^7^ These applications illustrate the framework’s versatility and relevance for structuring and communicating the multifaceted offerings of clinical informatics fellowships. When applied to clinical informatics fellowships, the PPT framework offers a low-burden, high-impact tool for improving transparency, comparability, and alignment between applicants and programs (Table 1).

**Table 1.** People, process, technology framework and clinical informatics fellowship.

People	Process	Technology and infrastructure
Mentorship and Organizational Influence	Research/Operational & Independence/Hands-On Support	EHR Type, Technical Skills, and Exposure to AI
Co-Fellows and Alumni Network	Clinical Work and Salary Structure	Remote, Hybrid, or On-Site Work Model
Board-Certified Faculty	Didactics, Master’s Courses, and Conferences	Laptops, Phones, Analytics Tools, and Branded Items
Cohort and Faculty Diversity	Number of Years Established	Geographical Setting and Cost of Living

## People: the human element of fellowships

The “People” aspect focuses on the individuals and communities that form the backbone of a fellowship. Fellowship candidates should examine the qualifications of both current fellows and mentors, while program directors must clearly convey these details. By focusing on the people, candidates can assess the social, professional, and academic environment they’ll be entering, while program directors can showcase their institution’s top-tier talent.

**Mentorship and organizational influence:** What roles do faculty play within the organization? Are the mentors involved in high-level strategic decisions? This information can signal mentorship approach and organizational impact.

**Co-fellows and alumni network:** The size and engagement of the co-fellow cohort play an important role in the learning experience. Fellowship programs with a strong alumni network can also offer invaluable job search support and open doors for future collaborations.^8^

**Board-certified informaticists:** How many faculty members are board-certified in clinical informatics, and in what clinical specialties? This can reveal the scope of opportunities within the fellowship.

**Cohort and faculty diversity:** The diversity, both demographic and experiential, of both the fellows and the faculty is essential. Exposure to various perspectives fosters collaboration and innovation.^9^ Applicants should ask about the l diversity of both current fellows and program leadership. Considerations should include access to partners in the diverse professional settings of research, operations, and industry.

## Process: how the fellowship operates

The “Process” dimension refers to the operational structure of a fellowship—how mentorship, learning, and project work are delivered. Transparency in this area helps applicants determine whether a program’s style aligns with their preferences and goals. Some fellowships emphasize academic research and self-direction, while others focus on operational work and offer robust hands-on guidance.

**Figure 1.**
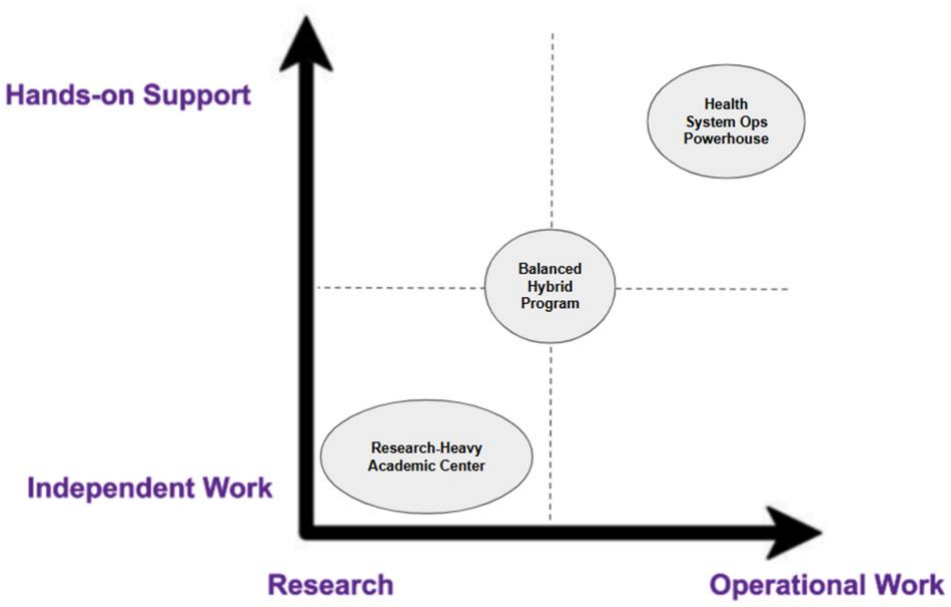
Research vs operations and independence vs hands-on support graph.

**Research vs operational and independence vs hands-on support:** Is the fellowship more focused on operational informatics or academic research, and what level of support and independence do fellows have? Figure 1 maps programs across two key axes:

- **X-axis (Research ↔ Operational Work):** 
  - *Research-focused* programs emphasize peer-reviewed publications, conference presentations, and protected time for academic inquiry.
  - *Operational-focused* programs prioritize implementation science, clinical decision support (CDS), EHR optimization, and quality improvement work embedded in health systems.
- **Y-axis (Independent Work ↔ Hands-on Support):** 
  - *Independent work* involves self-directed projects with a degree of autonomy in project ownership and execution within ACGME-compliant supervision structures.
  - *Hands-on support* includes scheduled mentorship meetings, scaffolded project development, and regular feedback from faculty or staff.

To make the framework more tangible, we’ve plotted three anonymized archetypes:

1. The Research-Heavy Academic Center
  - Strong emphasis on peer-reviewed publications and grant writing
  - Limited operational work
  - Fellows have significant independence
2. The Health System Ops PowerhouseDeep involvement in EHR implementation and QI projects
  - Minimal research expectations
  - Structured mentorship with embedded project teams
3. The Balanced Hybrid Program
  - Mix of research and operations
  - Moderate support with opportunities for independence

Applicants can use this grid to assess program fit, while program directors can use it to clarify their position and attract aligned candidates.

**Clinical work and salary structure:** How do fellows spend time clinically, and are they paid for it in addition to their fellowship salary? Programs that encourage paid clinical work may enhance the fellowship structure and financial flexibility by allowing fellows to integrate their informatics and clinical roles while earning additional income. Programs also vary in whether clinical work is performed within the same organization as the fellowship or permitted at external institutions—an important consideration for applicants evaluating overall workload and alignment with their training goals.

**Didactics, master’s courses, and conference funding:** Are structured educational offerings such as didactics, optional master’s programs, or funding to attend academic conferences available? Programs with robust didactics, access to interdisciplinary coursework, and financial means to support fellows at academic conferences ensure education requirements are met^10^ and give fellows the chance to tailor their learning experiences.

**Number of years established:** Established programs with a track record of successfully placing graduates into jobs in academia, industry, or healthcare systems offer a sense of stability. Program longevity can indicate institutional maturity, but newer programs may offer distinctive advantages—such as innovative training pathways, greater flexibility in curriculum design, and alignment with emerging institutional priorities that create immediate job opportunities for graduates.

## Technology: infrastructure and tools

In a field like clinical informatics, where technological tools play a pivotal role in day-to-day tasks, the “Technology” pillar is critical. Applicants should understand what technical training and resources they’ll have, while program directors should clearly outline the technological infrastructure in place.

**EHR type, technical skills, and AI exposure:** The type of electronic health record (EHR) system fellows are trained in significantly influences their technical skill set and future career opportunities. Programs that offer experience with EHR transitions, configuration, or optimization—and that support skill-building in tools like SQL, Python, R, or data visualization platforms—may provide a stronger foundation for technical leadership roles. These offerings should be clearly communicated in program materials.

As artificial intelligence (AI) becomes increasingly embedded in clinical workflows,^11^ exposure to AI tools is also a valuable component of training. This includes experience with clinical decision support systems, predictive analytics, and natural language processing. However, AI education should go beyond technical familiarity: fellows should be introduced to frameworks for ethical AI use, data governance, algorithmic transparency, and implementation science.^12^ Programs that incorporate these interdisciplinary perspectives will better prepare fellows to lead responsible AI integration in healthcare.

**Work setting:** Remote work or hybrid options are critical factors as they offer flexibility and insight into modern informatics roles that increasingly support decentralized workforces.^13^ Office spaces can also be invaluable when they allow for a comfortable workplace with opportunity to collaborate with team members.

**Resources provided:** The provision of resources such as laptops, phones, or other technology, as well as items like branded jackets or backpacks, also conveys a program’s commitment to its fellows’ success and can build capacity and morale.

**Geographical setting and cost of living:** Is the program in an urban, suburban, or rural environment, and how does this impact social opportunities and cost of living during fellowship? Applicants should consider these factors when assessing programs.

## Implications for program directors

The PPT framework offers valuable strategic utility for program directors. By applying the framework internally, programs can better define their strengths, identify growth areas, and improve alignment with prospective fellows.

**1. Program self-assessment and strategic planning** 

Program leaders can evaluate and communicate their offerings across the three key domains:

- **People**: Who are the mentors? What is the depth and breadth of faculty expertise? How diverse is the trainee and leadership cohort?
- **Process**: How is the fellowship structured? What balance of research, operations, and education is offered? What degree of independence is expected?
- **Technology and Infrastructure**: What systems (eg, EHRs, analytic platforms, AI tools) do fellows engage with? What technical training is provided?

**2. Curriculum development and continuous improvement** 

Programs can use this framework to ensure they are developing fellows across a balanced set of competencies. For example, if a program plots heavily on the “Operational/Hands-On” quadrant of the Research/Operations Grid, it may want to incorporate more academic mentorship opportunities or data science exposure to support fellows with research-oriented aspirations.

**3. Targeted marketing and recruitment** 

The framework also supports more transparent and effective communication with prospective applicants. Program directors can tailor website content, brochures, and interview-day presentations to attract fellows who are well-aligned with the program’s strengths and structure. This improves match quality and helps applicants make more informed decisions earlier in the application cycle.

## Sample analysis

To demonstrate the practical utility of the PPT framework, we conducted a preliminary analysis of 18 randomly selected clinical informatics fellowship program websites. The websites were selected from the AMIA-accredited fellowship website list as of July 2025. Each website was evaluated across 12 categories aligned with the PPT framework using a standardized coding instrument (Table 2). We assigned scores of 0, 1, or 2 to each category: 0 indicated no information present, 1 indicated some information present, and 2 indicated robust information present. Two authors (JS, PS) independently reviewed and scored each website using the proposed rubric, resolving any discrepancies by discussion and consensus to ensure consistency and transparency in the illustrative analysis. This simple rubric enabled us to systematically assess the accessibility and depth of information made available to prospective applicants, and to identify common gaps across programs (Table 3).

**Table 2.** Scoring rubric for clinical informatics fellowship website evaluation using the people, process, and technology (PPT) framework.

Category	0 = No Information	1 = Some Information	2 = Robust Information
**Mentorship and Organizational Influence (People)**	No mention of mentors or institutional leadership support.	Mentions 1-2 faculty or mentorship in general terms.	Clearly outlines named mentors, leadership roles, and how they are embedded in organizational decision-making.
**Co-Fellows and Alumni Network (People)**	No mention of current fellows or alumni.	Mentions existence of fellows or alumni.	Lists or profiles of current fellows and alumni, including their roles or outcomes.
**Board-Certified Faculty (People)**	No info on whether faculty are CI-boarded.	Mentions some CI-certified faculty.	Multiple named faculty explicitly listed with CI board certification.
**Cohort and Faculty Diversity (People)**	No mention of DEI or diversity in faculty/fellows.	Mentions commitment to professional and demographic diversity.	Emphasizes diversity of professional opportunities and/or demographic details.
**Research/Operational & Independence/Hands-On Support (Process)**	No description of fellow work or project structure.	Vague or brief mention of projects or operations.	Specific examples of projects, research opportunities, or fellow autonomy described.
**Clinical Work and Salary Structure (Process)**	No info on clinical duties or salary.	Mentions part-time clinical work or compensation vaguely.	Clear breakdown of clinical role (eg, 20% FTE) and transparent salary/benefits info.
**Didactics, Master’s Courses, and Conferences (Process)**	No info on coursework or training structure.	Mentions some training or educational resources.	Detailed curriculum including courses, certifications, degrees, or conference support.
**Number of Years Established (Process)**	No mention of how long program has existed.	Implies experience without stating timeline.	States year established or number of years of program history.
**EHR Type, Technical Skills, and Exposure to AI (Technology)**	No info on EHR or technical training.	Mentions an EHR vendor or general exposure to tools.	Details of EHR, coding training, analytics, AI exposure, or certifications.
**Remote, Hybrid, or On-Site Work Model (Technology)**	No info on work arrangement.	Implies in-person or mentions virtual meetings.	Clearly defines work model and physical building locations/amenities.
**Laptops, Phones, Analytics Tools, and Branded Items (Technology)**	No mention of resources provided to fellows.	Mentions IT access or analytics tools.	Describes laptops, phones, specific platforms/tools provided, or program-branded materials.
**Geographical Setting and Cost of Living (Technology)**	No info on location or environment.	Mentions city or region.	Discusses local lifestyle, housing, affordability, or urban/rural setting in detail.

**Table 3.** Anonymized summary of scores across 18 clinical informatics fellowship websites.

Category	Sub-Category	Score: 0	Score: 1	Score: 2
**People**	Mentorship and Organizational Influence	0	7	11
	Co-Fellows and Alumni Network	2	8	8
	Board-Certified Faculty	0	2	16
	Cohort and Faculty Diversity	2	16	0
**Process**	Research/Operational & Independence/Hands-On Support	0	6	12
	Clinical Work and Salary Structure	0	7	11
	Didactics, Master’s Courses, and Conferences	0	1	17
	Number of Years Established	2	5	11
**Technology**	EHR Type, Technical Skills, and Exposure to AI	0	8	10
	Remote, Hybrid, or On-Site Work Model	10	6	2
	Laptops, Phones, Analytics Tools, and Branded Items	3	14	0
	Geographical Setting and Cost of Living	0	2	16

This illustrative analysis is presented not as a formal research study, but as a practical demonstration of how the PPT framework can be applied to identify gaps and opportunities in publicly available program information. Our analysis revealed notable variation in the transparency and comprehensiveness of publicly available information. Most websites did a commendable job listing faculty members, often including names, credentials, and titles. However, few programs explicitly connected these faculty to specific projects, mentorship roles, or collaborative opportunities available to fellows—limiting applicants’ ability to assess mentorship fit. Conversely, lifestyle and geographic context were consistently well represented, with many programs describing the local community, cost of living, and proximity to major cities in detail. Didactic coursework was also a strength: nearly all sites offered robust descriptions of formal training opportunities, including graduate courses, certifications, and conferences. In contrast, information on tangible resources—such as laptops, analytics tools, or access to software—was sparse. Similarly, technical skills training (eg, Python, SQL, EHR builder certifications) was rarely detailed, despite its potential importance to prospective applicants. Additionally, few programs described physical facilities or amenities relevant to informatics work. These gaps highlight an opportunity for programs to better align their online presence with the practical and academic concerns of applicants, and demonstrate the value of structured evaluation tools like the PPT framework.

## Conclusion

In applying the People, Process, and Technology framework to clinical informatics fellowships, applicants gain a structured way to compare programs, while directors can better communicate their offerings. The fellowship landscape may benefit from more standardized program descriptions that emphasize these three pillars. Future research could focus on surveying fellows or program directors through this framework to build a comprehensive map of clinical informatics fellowship programs. By emphasizing this structured approach, applicants can ensure they are entering a program that matches their career goals, while directors can better attract the right candidates for their unique offerings.

## Data Availability

The data underlying this article will be shared on reasonable request to the corresponding author.
